# An adaptive non-local means filter for denoising live-cell images and improving particle detection

**DOI:** 10.1016/j.jsb.2010.06.019

**Published:** 2010-12

**Authors:** Lei Yang, Richard Parton, Graeme Ball, Zhen Qiu, Alan H. Greenaway, Ilan Davis, Weiping Lu

**Affiliations:** a Department of Physics, Heriot-Watt University, Edinburgh EH14 4AS, UK; b Department of Biochemistry, The University of Oxford, Oxford OX1 3QU, UK

**Keywords:** Denoising, Particle detection, Feature extraction, Non-local means filter, *Drosophila*, Microtubules

## Abstract

Fluorescence imaging of dynamical processes in live cells often results in a low signal-to-noise ratio. We present a novel feature-preserving non-local means approach to denoise such images to improve feature recovery and particle detection. The commonly used non-local means filter is not optimal for noisy biological images containing small features of interest because image noise prevents accurate determination of the correct coefficients for averaging, leading to over-smoothing and other artifacts. Our adaptive method addresses this problem by constructing a particle feature probability image, which is based on Haar-like feature extraction. The particle probability image is then used to improve the estimation of the correct coefficients for averaging. We show that this filter achieves higher peak signal-to-noise ratio in denoised images and has a greater capability in identifying weak particles when applied to synthetic data. We have applied this approach to live-cell images resulting in enhanced detection of end-binding-protein 1 foci on dynamically extending microtubules in photo-sensitive *Drosophila* tissues. We show that our feature-preserving non-local means filter can reduce the threshold of imaging conditions required to obtain meaningful data.

## 1 Introduction

Fluorescence live-cell imaging is commonly used to study intracellular molecular dynamics (Stephens and Allan, 2003), but only relatively recently has the full quantitative value of such data been commonly exploited (Jaqaman et al., 2008; Meijering et al., 2006). In live cell microscopic imaging there is always a compromise between image quality and cell viability. Excitation of fluorescent probes causes photo-bleaching and photo-toxicity, which limit the light intensity and exposure times that can be used. The requirement to image fast and in multiple dimensions to capture dynamic intracellular events also constrains illumination and exposure regimes and requires fast camera readout. This in turn results in low signal-to-noise ratio (SNR) fluorescence images with mixed Poisson–Gaussian noise (Stephens and Allan, 2003; Jaqaman et al., 2008; Meijering et al., 2006). Under such circumstances, denoising techniques become a critical tool to improve quantitative analysis of these images in order to understand dynamic intracellular processes and their underlying mechanisms (Jaqaman et al., 2008; Meijering et al., 2006; Sbalzarini and Koumoutsakos, 2005; Genovesio et al., 2006).

Mean filtering of adjacent pixels is one of the simplest tools for reducing noise contamination, but suffers from the disadvantage that resolution is reduced. A more intelligent approach to noise removal is to make use of the redundancy in images arising from repeated patterns and average only similar pixels. A popular implementation of this idea is the non-local means (NLM) filter (Buades et al., 2005; Buades et al., 2008), which estimates the coefficients for averaging (the extent to which pixel values may be averaged together) by comparing the similarity of patches of pixels. The NLM filter belongs to a class of filters known as feature-preserving filters. In addition to the NLM filter and its recently improved versions (Brox et al., 2008; Mahmoudi and Sapiro, 2005; Tasdizen, 2008; Yang and Clausi, 2009; Kervrann and Boulanger, 2008; Boulanger et al., 2008; Boulanger et al., 2010) the bilateral filter (Tomasi and Manduchi, 1998; Jiang et al., 2003; Pantelic et al., 2006) is also based on feature preservation. All of these methods attempt to identify features to be preserved while eliminating the noise by averaging.

In contrast to the feature-preserving approach, the alternative, edge-preserving approach, uses local information in images, namely the gradient of pixel gray values, to preserve the edges that bound image features. The partial differential equation and variational methods belong to this category (Perona and Malik, 1990; Rudin et al., 1992; Frangakis and Hegerl, 2001; Chan et al., 2001). In live cell fluorescence imaging, objects of interest are often small particles with limited pixel resolutions so that their edges are poorly defined and their detection can be easily compromised by noise (Meijering et al., 2006; Boulanger et al., 2008). Edge-preserving algorithms based on local gradient information may not, therefore, be robust in identifying the boundaries of small particle features to permit successful denoising of such images. On the other hand, a particle-like feature corresponds to a (small) localized area of high value pixels on average (Jaqaman et al., 2008; Sbalzarini and Koumoutsakos, 2005; Genovesio et al., 2006). This feature can be adopted to assist in noise reduction in feature-preserving approaches.

Recent improvements to the NLM filter have focused on more accurate and faster computing of the Euclidean distances (intensity differences) between neighborhoods, used to estimate the coefficients for averaging (Brox et al., 2008; Mahmoudi and Sapiro, 2005; Tasdizen, 2008; Yang and Clausi, 2009). The patch-based filter (PBF) (Kervrann and Boulanger, 2008; Boulanger et al., 2008; Boulanger et al., 2010; Carlton et al., in press) is a state-of-the-art development of the non-local means filter in which the sizes of the searching windows are adaptively selected, so achieving a better balance between the accuracy of the point-wise estimator and stochastic error at each spatial position. It has demonstrated a great capability for restoration of natural images with minimal *a priori* knowledge.

In this paper, we extend the NLM approach by employing an additional new and robust non-local statistic measurement to denoise low SNR fluorescence live-cell images. Our work is motivated by the observation that denoising algorithms based solely on the Euclidean distance measurement of pixel gray values (as in the existing non-local means approaches) may lead to poor feature preservation when they are applied to low SNR images containing weak particle-like objects. In our newly proposed method we adaptively apply the NLM filter to preserve these weak features using an additional term for the probability that a given pixel belongs to a particle. We show that our proposed feature-preserving method achieves an improved balance between feature enhancement and background smoothing. Moreover, it requires no precise information about noise distribution and is computationally efficient. In our experiments we establish that the method can achieve a higher peak signal-to-noise ratio (PSNR) and higher sensitivity to pick out true particles than several commonly used algorithms when applied to both synthetic and real fluorescence live-cell images. We first present the method underlying the new feature-preserving non-local means (FP-NLM) approach, including the construction of the particle probability images from original grayscale images. Tests of the FP-NLM filter are then carried out on synthetic data, sub-resolution bead images and live-cell images. Conclusions and remarks are presented at the end of the paper.

## 2 Method

**Fig. 1:**
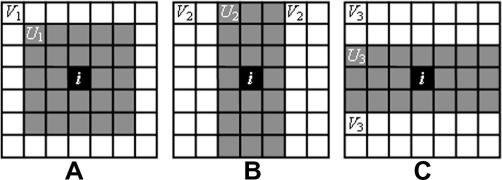
Three Haar-like features used to classify each pixel into two classes. (A) $H_{1}^{s}$; (B) $H_{2}^{s}$; (C) $H_{3}^{s}$, where *s* is the scale. For a central pixel *i*, *U*_*k*=1–3_ and *V*_*k*=1–3_ are the corresponding particle and background areas, respectively.

Visually, an important manifestation of a particle-like feature (particle) in live cells is a locally concentrated cluster of pixels with relatively higher gray values compared to their immediate surroundings. Our idea is to find a new non-local statistic method that can enhance such particle-like features in images that suffer from severe noise contamination and employ it for image processing. To achieve this goal, we have mapped an original grayscale image to a feature image that describes particle probability by novel use of (non-local) Haar-like features (Viola and Jones, 2004), which have proven very effective in human face detection algorithms. The image mapping is carried out in the following steps. First, the Haar-like feature calculation is made for each pixel. This calculation effectively determines the maximum contrast between a (scalable) small area centered at this pixel and the area of its immediate surroundings (Fig. 1A–C). A simple threshold is then applied to this value to classify the pixel into two classes, ‘likely belonging to a particle’ or ‘background’. A ‘particle probability’ at each pixel is then computed as the ratio of the number of pixels that likely belong to a particle and are also spatially connected, to the total number of pixels in a small region centered at the pixel. An adaptive non-local means filter is proposed which makes use of the new particle probability image and includes two Gaussian weighted Euclidean distance measurements: the first measures the similarity of pixel gray values between two neighborhoods in the original grayscale image (as in non-local means filter), whereas the second quantifies the similarity in particle probability between the same neighborhood. Since the latter measurement is capable of greatly enhancing the particles that are barely visible in the former, the proposed adaptive non-local means filter is intended to preserve weak particle-like objects when it is used to denoise low SNR fluorescent live-cell images.

### 2.1 Haar-like features

Haar-like features were first introduced to detect the human face. In the original work by Viola and Jones (2004), a very large set of simple rectangular features based on Haar functions (or wavelets) (Gonzalez and Woods, 2002) were constructed to measure the contrast between the facial features and their immediate surrounding areas, i.e., the difference between the average pixel gray value of the feature versus its neighborhood. In fluorescence live-cell images a particle has, on average, higher pixel gray values than its immediate surroundings (background), even in the case of poor SNR and contrast. We therefore use three simple Haar-like features $H_{k}^{s}$ (*k* = 1, 2, 3) to detect particles in living cells, where the index *s* denotes the scales (size) of the features. As depicted in Fig. 1, for a given scale the shaded (particle) areas have different shapes or orientations and are surrounded by the white (background) areas within a square Haar window. The features are measured by:

(1) $$H_{k}(i)=\underset{s}{\max}(H_{k}^{s}(i))\equiv \underset{s}{\max}\{M_{U_{k}}(i\text{,}s)-M_{V_{k}}(i\text{,}s)\}$$

where $M_{U_{k}}$ and $M_{V_{k}}$ are respectively the means of the pixel values in the shaded area, *U*_*k*_, and in the white area, *V*_*k*_, within the Haar windows centered at pixel ***i*** (***i*** ≡ (*x*,*y*)). Therefore, *H*_*k*_ is the maximum difference at different orientations of the averaged gray values between a local area and its surroundings for different scales (sizes of Haar windows). These features can be considered as a set of multi-scale directional steerable filters (Freeman and Adelson, 1991), which were designed to obtain optimal response of a filter at different orientations and scales. In applying Eq. (1), the sizes of the objects of interest are first estimated empirically from the raw image data, and the maximum size of the shaded area in the Haar window should be set just larger than that of the objects. Since features in live cells are usually much simpler than those of a human face, no spatial correlation of different Haar-like features is required to identify the particle-like objects. Consequently, the decision network used in the face detection is not required. Instead, we combine the three Haar-like features linearly:

(2) $$\overset{¯}{H}(i)=\overset{3}{\underset{k=1}{\sum }}c_{k}H_{k}(i)$$

where *c*_*k*_,$\sum_{k}c_{k}=1$, are the weights of the Haar-like features. For objects of no preferred shape and orientation, an equal weight of *c*_*k*_ = 1/3 (*k* = 1, 2, 3) is the obvious choice. In order to use the Haar-like features in a simple manner to construct a particle probability image, we classify each pixel, ***i***, to belong to a particle if:

(3) $$\overset{¯}{H}(i)⩾\lambda$$

where *λ* is the (positive) threshold value in determining if the grayscale difference value is consistent with a region being a particle or not. In applying Eq. (3) we empirically choose a ‘weak’ threshold value, *λ*, so that few pixels belonging to a particle are wrongly excluded (see Table 1). Subsequent steps are able to further reduce false positives as described below.

### 2.2 Particle probability image

We use the results of Eq. (3) to construct a particle probability image from the original grayscale image. To do so we define the probability of finding a particle at position ***i*** to be the ratio:

(4) $$P(i)=(\Delta N/N_{\mathrm{tot}}){\;}_{A_{i}}$$

where *N*_*tot*_ is the total number of pixels in a given (small) area *A*_***i***_ centered at ***i*** and Δ*N* is the number of pixels in *A*_***i***_ that satisfies $\overset{¯}{H}(i^{\prime })⩾\lambda$ (including ***i***′ = ***i***) and are spatially connected. The number Δ*N* is calculated by using the simple 4-nearest neighborhood growing strategy (Gonzalez and Woods, 2002) from the center of the neighborhood. The particle probability image is generated by applying Eq. (4) at each pixel position and therefore depends upon: the Haar-like feature at this pixel, those in the surrounding area and whether these pixels are spatially connected. The probability is high only when there is a concentration of pixels that satisfy Eq. (3), which reflects very well the characteristics of a particle in a grayscale image. Initial overestimation of the particle class (those pixels that belong to particle features), due to the weak threshold introduced in Eq. (3), is reduced by application of this connectivity criterion, since noise spikes are randomly distributed in space and are not spatially connected. In general, the size of the neighborhood in measuring Eq. (4) is chosen to be close to the size of the smallest particle expected in an image.

**Fig. 2:**
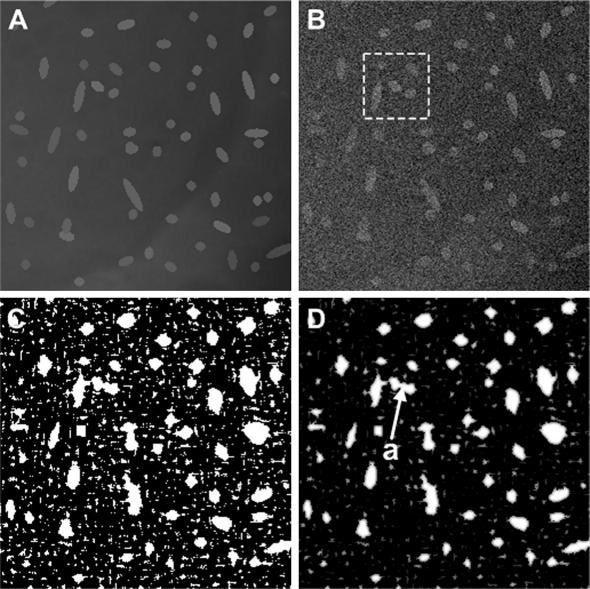
Mapping of a grayscale image to a particle probability image. (A) Synthetic noise-free image; (B) corresponding noisy image (standard deviation *σ* = 20 for 8 bit data); (C) binary image showing classification results, the 5 Haar-like window sizes used were 7 × 7, 11 × 11, 17 × 17, 25 × 25, 37 × 37 (increment of 1.5 times), and *λ* = 7.0 based on the 20% threshold criterion; (D) particle probability image.

We undertook a case study mapping a grayscale image to a particle probability image by using Eqs. (1)–(4) on a synthetic image that simulates live-cell data recorded from a microscope. The data are constructed by using a linear model (Boulanger et al., 2008; Boulanger et al., 2010) comprising particles, uneven background, Poisson and Gaussian noises (standard deviation *σ* = 20 for this 8 bit data). The synthetic noise-free and noisy images are shown as Fig. 2A and B. As seen, particle-like features have varying signal strengths and shapes, the size of which can be estimated by using the two (orthogonal) axes of a particle, the shortest is around 5 pixels whereas the longest is 30 pixels. Accordingly, we choose 5 different Haar-like windows (*s* = 1–5), from 7 × 7 to 37 × 37 pixels, to ensure that the shaded areas in the Haar window varies sufficiently to cover the size range of the particles as required for measuring Eq. (1). To allow for a sufficient margin to detect faint particles in images, we empirically set the threshold *λ* in Eq. (3) to be 20% of the difference in the averaged pixel gray values between a typical particle region and a typical background region across the imaged field. Setting the threshold value in this way avoids falsely excluding true particle objects and accommodates particle intensity variations across the images under investigation. We find that this threshold determination works well for the synthetic and real live-cell images presented in this paper. [Note: with images where signal falloff is severe, image data should first be flat field corrected (for example, by using adaptive histogram equalization methods (Pisano et al., 1998; Stark, 2000)) before applying Eqs. (1)–(4).]

A binary image from Haar-like feature based classification is presented in Fig. 2C (bright for $\overset{¯}{H}(i)⩾\lambda$ and dark for $\overset{¯}{H}(i)<\lambda )$ showing that all the particles in the image are identified. The isolated high gray value pixels are a consequence of overestimation due to the weak threshold and high noise contamination. Fig. 2D is the particle probability image derived from the binary image by using Eq. (4) with *A*_***i***_ being 5 × 5 pixels, which corresponds to the size of smallest particles in the image. By comparison to the noise-free image (Fig. 2A), we find the distributions and strengths of the probabilities (Fig. 2D) are generally well matched to the real particles in the grayscale image. While the algorithm at this stage does still overestimate the identification of particles in this image, the effect is reduced in comparison to the binary image (Fig. 2C) because the isolated spikes in the latter are averaged over the area of the neighborhood. As seen in Fig. 2D (marked by ‘a’), the overestimation due to noise and limited resolution of the Haar windows used can lead to the joining together of two or more spatially proximate particles in the probability image. We show below that the overestimation at this level does not play an important role in denoising in our proposed feature-preserving non-local means (FP-NLM) filter.

### 2.3 Feature-preserving non-local means (FP-NLM) filter

In the newly proposed FP-NLM filter an image is processed by two Gaussian weighted Euclidean distances, measured in grayscale and particle probability images. As in the conventional NLM filter, the processed gray value at pixel ***i*** of image *F*, *FPNLM*(*F*)(***i***), is given as the weighted average of all pixel grayscale values in a searching window *W*_***i***_ centered at ***i***:

(5) $$\mathrm{FPNLM}(F)(i)=\underset{j\in W_{i}}{\sum }\omega (i\text{,}j)F^{\prime }(j)$$

where *F*′(***j***) = *F*(***j***) if there is no pre-processing on the original image. The weight function $\{\omega (i\text{,}j)\}{\;}_{j\in W_{i}}$ in Eq. (5) is in a non-local Gaussian form of:

(6) $$\omega (i\text{,}j)=\frac{1}{A(i)}\exp\left(-\frac{{\left\|V(N_{i})-V(N_{j})\right\|}_{2\text{,}a}^{2}}{h^{2}}-\frac{{\left\|P(N_{i})-P(N_{j})\right\|}_{2\text{,}a}^{2}}{g^{2}}\right)$$

where **V**(*N*_***i***_) is the vector of the pixel gray values taken from the neighborhood *N*_***i***_ centred at ***i*** and **P**(*N*_***i***_) is the vector of the particle probability values from the same neighborhood, the Euclidean distance ${\left\|-\right\|}_{2\text{,}a}^{2}$ is a classical *L*_2_ norm, convolved with a Gaussian kernel of standard deviation *a* (Buades et al., 2005). The first term is therefore the Euclidean distance of pixel gray values between the two neighborhoods *N*_***i***_ and *N*_***j***_ within the searching window, as in the conventional NLM filter. Similarly, the second term is the Euclidean distance of particle probabilities between the two same neighborhoods taken from the probability image. The sizes of the neighborhood and searching window can be chosen by following the same criteria in NLM filter. The parameters *h* and *g* control the strengths of the first and second weights in Eq. (6), the values and ratio of which must be set appropriately (see Table 1) in order to achieve a good balance between background smoothing and object enhancement, *A*(***i***) is the normalization factor ensuring $\sum_{j\in W_{i}}\omega (i\text{,}j)=1$. When *g* → ∞, the second term in Eq. (6) can be dropped and the FP-NLM filter is reduced to the conventional NLM filter (Buades et al., 2005), in which the weighted averaging is determined by only the first term that measures the Euclidean distance from the grayscale image *F*′. The second term can therefore be regarded as an adaptively varying coefficient that controls the filtering strength of the conventional NLM filter (first term). In low SNR environments with non-Gaussian noise, when the Euclidean distance measurement fails to establish a difference between a particle region and background region in a grayscale image, the first term attempts to use the background information to smooth the particle region. However, the same measurement in the corresponding particle probability image behaves very robustly because particle features are well preserved in this image. Consequently, the second term acts to protect the particle region by using the probability information to reduce the filtering strength of the first term. On the other hand, isolated spikes in the probability image due to overestimation will not survive because there is little signature of particles in the original grayscale image. The choice of *h* and *g* also depends on whether the goal is to denoise the image or to improve detection. The former (denoising) usually requires a balanced filtering on both feature and background areas whereas the latter (particle detection) pays particular attention only to feature recovery. For the latter case, we can increase the second weighting (reduce *g*) in Eq. (6) to improve the detection of weak features in low SNR images. A more detailed discussion is given in the experimental Sections 3 and 4. Moreover, since each pixel in the original image *F*(***j***) has been classified using Haar-like features into two classes by Eq. (3) and few pixels belonging to the particle class have been wrongly classified due to the use of a weak threshold value, a simple pre-processing of *F*(***j***), such as mean filtering the background region before applying Eq. (5), may be useful. A large mean filter window can heavily smooth the background while a small one can remove undesirable high frequency components. The choice of this window size depends on the images under investigation and the biological application. We show later in experiments on synthetic images that this simple mean filtering can significantly reduce artifacts in background that may be caused by the subsequent non-local means filtering.

In summary, the proposed algorithm for FP-NLM filter works in three steps. First: calculation of Haar-like features, initially calculated in Eq. (1) and then combined using Eq. (2), followed by binarization according to the threshold condition Eq. (3). Second: construction of the particle probability image by using the definition Eq. (4), which is the ratio of the number of pixels that satisfy Eq. (3) and are spatially connected to the total number of pixels in a selected small area. Third: generation of a denoised image in the form of the Gaussian weighted average Eq. (5) in which the coefficient is given by Eq. (6), which is based on two Euclidean distance measurements in the grayscale and particle probability images.

## 3 Testing algorithm performance with synthetic data

**Fig. 3:**
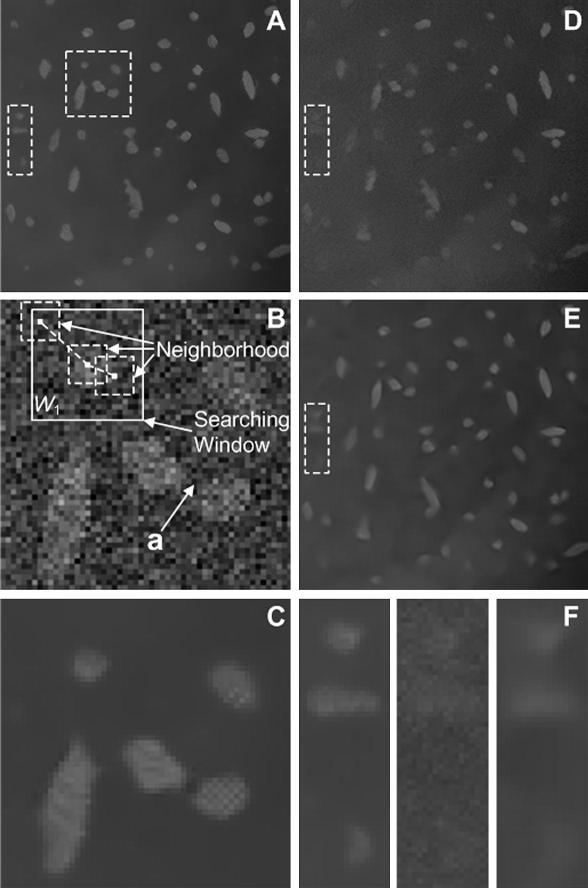
Tests on the synthetic noisy image shown in Fig. 2B. (A) FP-NLM filtered; (B) a selected image region taken from Fig. 2B and enlarged; (C) FP-NLM filtering result in the selected region; (D) denoised image of Fig. 2B by NLM filter; (E) denoised image of Fig. 2B by PBF; (F) denoised images of the area marked by the white box in (A, D, and E) shown enlarged, left: FP-NLM filtered, middle: NLM filtered and right: PBF filtered. The filtering parameters are given in the main text.

We first tested the FP-NLM filter (Eqs. (5) and (6)) on the synthetic image shown in Fig. 2B. The neighborhood was set to be 7 × 7 pixels whereas the searching window is 21 × 21 pixels, both of which follow the suggested values for NLM filter (Buades et al., 2005) for a good balance between the performance and the computational cost (see Table 1). The first filtering parameter *h* determines the conventional non-local means averaging weight, the value of which is chosen within the operation window of *h* = *α*_1_ · *σ* with *α*_1_ ∈ [0.75,1] for a ‘high visual quality solution’ (Buades et al., 2008). The second filtering parameter *g* determines the level of enhancement in the particle regions. We found that, for the given values *h*, an operation range of *g* = *σ*/*α*_2_ with *α*_2_ ∈  [50,100] gives empirically a good balance between background smoothing and particle enhancement in denoised images. Since both parameters are proportional to *σ*, their relative strengths remain the same for varying noise levels in images. The large difference of the two coefficients (1/*α*_2_ and *α*_1_) is due to the fact that the particle probabilities are normalized but gray values are not. We chose a middle value of *h* = 0.9*σ* and *g* = *σ*/70 for the present image under investigation. The denoised image by FP-NLM filtering is shown in Fig. 3A, where *F*′(***j***) in Eq. (5) was obtained by applying a mean filter of 5 × 5 pixels to *F*(***j***) in background regions classified by the binary image Fig. 2C (black pixels). Fig. 3A has correctly identified all particles by comparing to the noise-free image Fig. 2A, including those which are barely visible in the noisy image Fig. 2B.

**Fig. 4:**
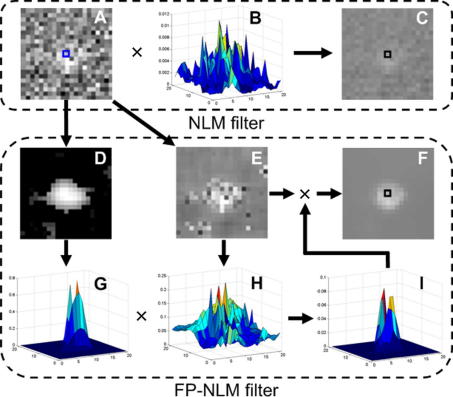
Comparison of the denoised results for the central point (marked by a small box in A, C, F) of the searching window between FP-NLM and NLM filters. Upper box – NLM filter: (A) a selected particle region from *W*_1_ of Fig. 3B, (B) normalized weights and (C) denoised result. Lower box – FP-NLM filter: (D) particle probability image and (G) associated weights, (E) pre-processed image of *W*_1_ and (H) associated weights, (I) normalized weights and (F) denoised result. Pixel values in (A), (C), (E) and (F) have been increased by 2 times for better display. The parameters are the same as used for Table 2, and given in the main text.

To characterize how the FP-NLM filter performs, we analyzed in detail the results for a small image area (Fig. 3B), taken from the square window in Fig. 2B, and compared to those by NLM filter which is a special case of FP-NLM filter when the second weight in Eq. (6) is neglected (*g* → ∞). We first looked into the region marked by a search window in Fig. 3B. The results of the NLM filter are given in the upper box in Fig. 4, where the gray value at the center of the denoised image (C) is the weighted average of the Euclidean distance (B) on the grayscale image (A) over the search window. Since the weights (B) are dispersed over the region due to noise contamination, the enhancement of the central pixel in (C) is weak. The FP-NLM filtering process is shown in the lower box, where the probability image (D) gives rise to strong weighting (G) in the particle area, whereas the pre-processed image (E) (background smoothed version of the original image (A)) leads to a weighting profile (H) which is similar to that obtained by NLM filter (B) in the particle region but reduced in the background region due to the pre-processing. The overall weights (I) are the product of the two functions (G) and (H), showing a strong enhancement to the central (particle) region. As a result, the denoised image (F) has a higher contrast compared to the image (C). Moreover, the same argument can explain how the errors due to particle overestimation in the probability image can be compensated by the correct measurement in the grayscale image. An example is pixel ‘a’ in Fig. 3B. While this pixel is incorrectly classified as belonging to a particle in the probability image (Fig. 2D), it is clearly part of background in the grayscale image (Fig. 3B). The two nearly touching particles are clearly distinguished as separate in the denoised image as shown in Fig. 3C.

**Fig. 5:**
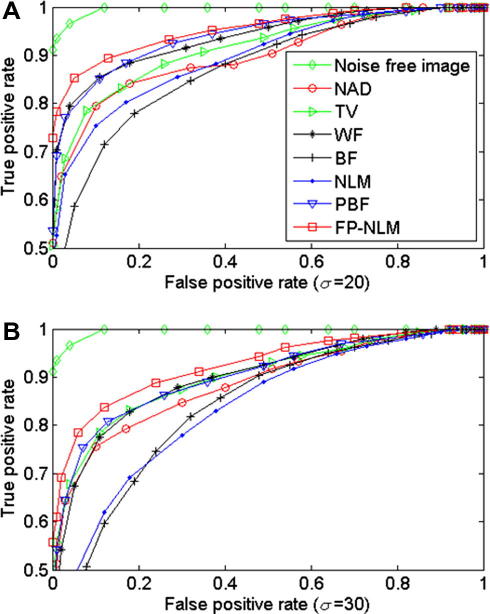
Comparison of receiver operating characteristic (ROC) curves for denoising results of different algorithms (see figure key) on the synthetic image shown in Fig. 2A with noise of standard deviations *σ* = 20 (A) and *σ* = 30 (B).

We show the denoised results of the same image by NLM filtering and PBF in Fig. 3D and E. The parameters in the NLM filter are the same as ours, whereas in PBF the patch window is 9 × 9, the maximum number of increments for the nested window size is 4, the critical parameters *λ*_0.01_ = 113.5 and *ρ* = 3, the reasons for choosing these parameter values for PBF are explained in the original papers (Kervrann and Boulanger, 2008). By comparing Fig. 3A–D and E, the FP-NLM filter and PBF perform noticeably better than the NLM filter. Strong noise contamination leads to over-smoothing of particles, particularly weak ones, using NLM and PBF filters, which can result in loss of potentially important information. This is shown in Fig. 3F displaying the denoising results by the three filters in the area marked by the box in Fig. 3A. Retaining the background context underlying particle features can be important to the interpretation of biological data. We find that FP-NLM filter also preserves well the gross non-particle features of the background (comparing Fig. 3A to Fig. 2A). This is primarily due to the use of simple mean average to pre-process the background of the original image, which reduces possible artifacts in the images when Eqs. (5) and (6) are applied after deconvolution. We can quantify image fidelity by calculating the peak signal-to-noise ratio (PSNR) (Kervrann and Boulanger, 2008) between the original and denoised images by FP-NLM filter and several commonly used filters: nonlinear anisotropic diffusion (NAD) (Perona and Malik, 1990), total variation (TV) minimization (Rudin et al., 1992), Wiener filtering (WF) (Gonzalez and Woods, 2002), bilateral filtering (BF) (Tomasi and Manduchi, 1998), NLM filtering and PBF (Kervrann and Boulanger, 2008). The NAD method is based on a heat (diffusion) equation that is capable of preserving edges while smoothing other areas. The diffusion function used here is (1 + (∣∇ *F*∣^2^)/*K*^2^)^−1^ with 150 iterations. The TV method tackles edge-preserving smoothing by minimizing an energy functional to achieve the best balance between the TV norm and image fidelity. We set the Lagrangian multiplier (Rudin et al., 1992) in the energy functional to be 0.05. WF reduces noise present in the image by comparison with the mean square error (MSE) estimation of the desired noiseless image. Here we use a window of 9 × 9 pixels to estimate local noise distribution for WF (Matlab function wiener2). BF is an improved method of Gaussian low-pass filtering which can prevent smoothing across edges. Here the filtering window size was set to be 31 × 31 pixels, and the standard deviations *σ*_*domain*_ = 5 and *σ*_*range*_ = 50. The results in Table 2 show that the FP-NLM filter achieves the highest PSNR. Moreover, since an important application for denoising fluorescence live-cell images is to facilitate detection of features of interest, we have computed the receiver operating characteristic (ROC) curves for the binarized denoised images as the threshold is varied (Fig. 5). Here the true positive rate (TPR, sensitivity) is the proportion of pixels belonging to particles that are correctly recognized and false positive rate (FPR, specificity) is the proportion of pixels belonging to background that are falsely classified, all measured against the known binary class of the noise-free image of Fig. 2A (representing the ‘ground truth’). The higher sensitivity achieved by FP-NLM filtering indicates its better capability to pick out true particles from images with different levels of noise contamination. These results indicate that our FP-NLM filter is the preferred choice to be used for improving particle detection.

## 4 Applying the algorithm to real data

At present, our ability to quantitatively determine the distribution and orientation of the individual microtubules (MT) that constitute a microtubule network is limited in various ways. Examination of markers in fixed material provides a static view and at best reveals only the gross distribution and net orientation. Tracking individual MT with end binding-protein 1 GFP (EB1-GFP) marking extending MT plus ends in living cells, can provide detailed information of dynamics, distribution and orientation (Stone et al., 2008). However, the use of EB1-GFP is hampered by difficulties in achieving images from live tissue of sufficient quality to track automatically, which limits our ability to analyze sufficient data to provide biologically and statistically relevant results.

In order to first evaluate the performance of our FP-NLM filter in a controlled situation, where we have a reliable ground truth and can easily manipulate the imaging conditions, we tested the filter on a field of sub-resolution fluorescent beads (Invitrogen, 200 nm NileRed beads). Thereafter, we applied our FP-NLM filter to live images of EB1-GFP and assessed its ability to achieve the required image quality for meaningful quantitative analysis. The results of EB1 tracking are outside the scope of this paper and will be published separately.

### 4.1 Test one

**Fig. 6:**
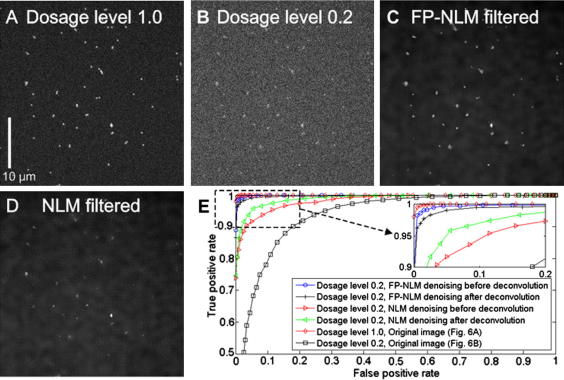
Tests of denoising algorithm performance on a field of sub-resolution fluorescent beads representing spot-like features. (A) Good SNR image (dosage level 1): deconvolved single Z plane. (B) Low SNR image (dosage level 0.2): deconvolved single Z plane. (C) Denoised result on Fig. 6B (PF-NLM filtered and then deconvolved). (D) Denoised result on Fig. 6B (NLM filtered and then deconvolved). (E) ROC curves comparing different denoising performances (see figure key). Note: images (A–D) have been individually contrasted for better display.

We tested the FP-NLM filter on a field of 200 nm fluorescent beads representing spot-like sub-resolution features (imaged on an OMX imaging system, from Applied Precision, with a Roper Cascade II back-thinned EMCCD detector in conventional mode). We took a range of exposures to analyze the capability of FP-NLM denoising to reduce the illumination threshold required to obtain meaningful results for analysis. We changed illumination dosage by varying exposure time and laser power. In this way we could achieve images of different signal-to-noise ratio (SNR). Normally, 1% laser power setting (with a 200 mW, 488 nm laser) for 10 ms achieves good image quality with fluorescent beads (Fig. 6A) and we refer to this setting as dosage level 1 (bead signal is measured 33 gray levels above a background level of 51 gray levels; background standard deviation is 6 gray levels, so giving SNR of 5.5). According to the Rose criterion (Rose, 1948), images with SNR ⩾ 5 are required for reliable feature detection. Fig. 6B shows the same slide as Fig. 6A but imaged at a lower illumination dosage with only bead intensity of 7 gray levels above the background. The dosage level for this image is 0.2 relative to the reference dosage of 1. The SNR of this image is 1.4. To improve particle detection in the dosage 0.2 image in Fig. 6B, we applied the FP-NLM filter to the raw data and then deconvolved the denoised image. The resulting image is plotted in Fig. 6C, which shows a good recovery of the spot-like features (SNR = 4.7) and background noise suppression compared to Fig. 6A and B.

We constructed a ‘ground truth’ for the bead data using a very high quality image (SNR = 19), by applying a simple detection threshold defined as the average of the maximal pixel gray value and the mean value of the image to unambiguously identify particles. We used this ground truth to measure the receiver operator characteristic (ROC) of the images shown in Fig. 6A–D. The ROC curves are shown in Fig. 6E. As can be seen, the ROC for image Fig. 6A at dosage level 1 (SNR = 5.5) has nearly perfect sensitivity and specificity, indicating that the spot-like features can be detected without applying any denoising algorithm. However, for the image in Fig. 6B at dosage level 0.2, the deterioration of the image quality is evident from the ROC, also shown in Fig. 6E, where significant errors occur when detection is performed on this image. The ROC of the denoised image is shown to be close to that of Fig. 6A, demonstrating that reliable detection has been achieved. Together, these results show that, by applying the FP-NLM filter, the illumination threshold required to obtain reliable particle detection (SNR approaching 5) can be reduced by a factor of almost five times for this data. In Fig. 6E we also plot the ROC of the denoised image obtained using the NLM filter (Fig. 6D, SNR = 2.7) for comparison. The curve is noticeably below that of FP-NLM filtered image, indicating less effective denoising.

Since wide field imaging coupled with deconvolution is often used for rapid imaging of live cells, we also used the bead data to compare the efficacy of denoising before deconvolution versus denoising after deconvolution. Each is known to have drawbacks (Chaux et al., 2007). In the former approach, denoising may modify the point spread function (PSF) model of the imaging system and change the noise statistics, both of which can lead to problems in the following deconvolution operation. Following the latter approach, deconvolution can lead to the propagation of noise in images and a change of its statistics, both of which pose difficulties for the subsequent denoising processing. The results are shown in Fig. 6E for both FP-NLM and NLM filters. We find that while the denoising before deconvolution shows a better result for the FP-NLM filter, denoising after deconvolution performs slightly better for the NLM filter as judged by the ROC curves (Fig. 6E). Over-smoothing is evident in the NLM denoised image (due to the low SNR of the raw data) and amplification of these errors upon deconvolution explains the poorer performance of this approach in the case of NLM filtered images. In summary, we find that the success of deconvolution before or after denoising depends on the denoising algorithms involved and that the difference between the two is modest in terms of particle detection as measured by ROCs.

### 4.2 Test two

**Fig. 7:**
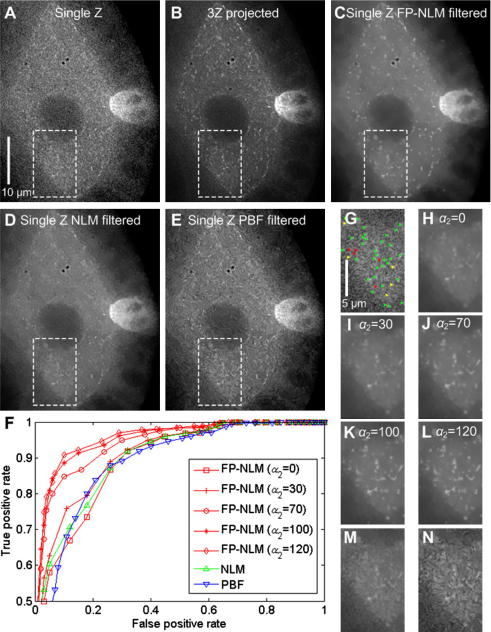
Application of denoising to the detection of EB1 foci in a *Drosophila* egg chamber expressing EB1-GFP. (A) Low SNR image: single time-point, single *Z*-plane. (B) Good SNR image: same time-point as (A) after average projection of 3 *Z*-planes (see main text). (C) Resultant image after denoising (A) by FP-NLM filter with *λ* = 10.0 (based on the 20% selection criterion), *h* = 0.9*σ*, *g* = *σ*/70 and *σ* = 20. (D–E) Resultant images after denoising (A) by NLM filter (D) and PBF (E). (F) ROC curves comparing filtering performance (see main text). (G) Manual identification of EB1 particles for the subregion marked in (A), arrowheads indicate the locations of particles, green: strong particle; yellow: weak particle; red: particle not discernible. (H–L) Denoised images of the subregion marked in (A) for the five different values of *g* = *σ*/*α*_2_tested [*α*_2_ = 0; 30; 70; 100 and 120, respectively, with a fixed value of *h* = 0.9*σ*] other parameters used are the same as in the experiments on synthetic image data. (M–N) Denoised images of the subregion marked in (A) using NLM and PBF, respectively. Scale bars A–E = 10 μm; G–N = 5 μm. (For interpretation of the references to colour in this figure legend, the reader is referred to the web version of this article.)

Our final test of the FP-NLM filter was on a live-cell image sequence of EB1-GFP, expressed in the *Drosophila* egg chamber where the MT cytoskeleton is complex and imaging is a considerable challenge (Fig. 7). In the *Drosophila* egg chamber, MT based transport is responsible for the localization of specific mRNA transcripts which in turn establish the polar axis of the developing fly (Becalska and Gavis, 2009). The egg chamber is therefore a good model in which to study the mechanisms involved in the determination of cellular polarity.

To test how well the algorithm performs in identifying particles under poor SNR conditions we validated the denoised image against a ground truth as with the bead data (see later). Image data was collected on a DeltaVision imaging system from Applied Precision (using a Roper Cascade II back-thinned EMCCD detector in conventional mode) over three *Z* planes (200 nm, spacing) with 30 ms exposures at 32% illumination intensity, (300 mW Xenon lamp light source), and then deconvolved with softWoRX Resolve 3D software (Applied Precision). Fig. 7A is a noisy image from a single Z plane at time-point 70 (of 120) to be used to test the FP-NLM filter – the EB1 foci (or particles) are barely visible. Fig. 7B is the corresponding ‘high quality’ image with good SNR generated by average projecting the three *Z* planes at that time-point. With increased SNR the EB1 foci are clearly visible.

The low contrast between particles and background from the cytoplasmic EB1-GFP signal in this type of image data (Fig. 7A) poses a serious challenge to denoising algorithms based solely on the Euclidean distance measurement of grayscale images. The additional information from the proposed feature image in the FP-NLM filter thus becomes crucial in processing these images. We first apply Eqs. (1)–(4) to construct the probability image where the threshold in Eq. (3) is set to be *λ* = 10.0 (calculated as 20% of the intensity difference between particle and background regions as described in Section 2.1) and then use (5) and (6), with parameters *h* = 0.9*σ* and *g* = *σ*/70, to obtain the denoised image, where the noise level is measured as *σ* = 20 on the raw image data. (Note that since deconvolution changes the noise statistics, the noise level of deconvolved data cannot be simply measured by standard deviation.) The denoised image is shown in Fig. 7C. As seen, particle-like objects have been significantly enhanced and noise in background regions has been suppressed, making the particles of interest readily identifiable. The overall structure of the *Drosophila* egg chamber is well preserved in the denoised image. By comparison, the NLM filter results in over-smoothing of many particles in the image due to the low contrast of these particles against a noisy background (Fig. 7D). When PBF is applied, particles of interest at different intensity levels have been enhanced (Fig. 7E), demonstrating the improved capability of the algorithm through adaptively varying the search window size and the filtering strengths. However, filtering of the background region has created obvious artifacts from the noise – compare Fig. 7N versus J and B: spurious swirling patterns are evident throughout the PBF result, which are noticeably absent from the corresponding FP-NLM result J. FP-NLM avoids this problem because the particle probability image provides additional vital information about particle presence, based on which, both particles and background are dealt with optimally.

Since the variations of parameters *h* and *g* in our FP-NLM filter can adjust the relative strengths between background smoothing and object enhancement as discussed earlier (Section 3), we make use of this capability to facilitate the improved detection of particles with lower contrast. The results are shown in Fig. 7H–L for the white-boxed subregion for different ratios of *g*(*g* = *σ*/*α*_2_, *α*_2_ = 0, 30, 70, 100, 120) to *h* (fixed at *h* = 0.9*σ*). Visual inspection of Fig. 7H–L reveals a gradual increase in particle clarity with the increase of the ratio *h*/*g*, the effect appears to saturate for *α*_2_ = 100 and further increase of *α*_2_ leads to over-enhancement of features. The parameter ranges for *h* and *g* are consistent with those given earlier. For comparison, the denoised images for the same subregion by NLM filtering and PBF are shown in Fig. 7M and N, respectively. As seen, FP-NLM filter performs noticeably better than PBF in particle enhancement and background smoothing.

To measure the effectiveness of the denoising algorithms tested, we used the ROC approach applied earlier (Fig. 5). The ground truth, representing true EB1 foci, was constructed for the highlighted subregion in the *Z*-projected image (Fig. 7B) by applying a low detection threshold for binarization. This essentially guaranteed no false negatives. False positives were then eliminated by their failure to match EB1 trajectories across the movie sequence. In this way, manual classification of all the detected pixels as particle or not particle was achieved. Fig. 7G shows corresponding foci identified in the low SNR image (Fig. 7A) where visible foci are marked by green arrows, barely visible ones are marked by yellow arrows and those which could not be identified are labelled in red.

Fig. 7F shows the ROC curves for Fig. 7H–N. As can be seen, the FP-NLM filter achieves a higher true positive rate with increasing value of parameter *α*_2_ up to *α*_2_ ≈ 100, after which the curves start to saturate. We note, based on Fig. 7F and H–L, that optimal parameters for particle detection and image denoising can be somewhat different. This is because the former focuses on the identification of particles whereas the latter requires a balanced recovery of both particle and background. Optimal denoising results for this case occur at around *α*_2_ = 70, whereas optimal particle detection occurs around *α*_2_ = 100 based on the ROC curves (Fig. 7F). Again, based on the ROC curves, we find that the FP-NLM filter is preferred to PBF and NLM filters for our biological applications to identify particles of interest in such low contrast images.

## 5 Discussion and conclusions

We have presented a new approach for denoising low SNR live-cell images, focused upon improving the identification of particle-like features. We have tested the new algorithm on simulated data, sub-resolution bead images and live-cell images and demonstrated good performance in enhancing particle contrast, reducing the background noise and minimizing artifacts. The algorithm performs consistently within the operation range of its parameters (see Table 1).

The key in our FP-NLM approach is the calculation of an additional similarity measurement representing ‘particle feature probability’ in the original grayscale image, the inclusion of which leads to significantly enhanced particle contrast. We use Haar-like features to estimate rather than precisely determine the presence of particles at the pixel level. Because it is an estimate only, the simple threshold condition given by Eq. (3) is an effective approach chosen to construct the particle probability. This is a robust measure because it depends not only on the classification at each pixel but also their spatial connectivity. The particle probability image constructed in this paper is especially designed for enhancing weak, discrete particles and for this purpose it improves the performance of FP-NLM compared to existing NLM and PBF.

Our approach is generally applicable for different feature sizes and shapes, since an unlimited variety of Haar-like features can be constructed to extend the basic NLM filter. We have tested various other Haar-like features and found that the three features with different scales of Haar windows that we have selected are a simple but effective choice. A single Haar-like feature (for example $H_{1}^{s}$ in Fig. 1A) can work adequately if the particle-like features under investigation have a round shape, whereas inclusion of more Haar-like features than the current three, for example by constructing two additional features with diagonally shaded regions in Haar windows, may increase classification precision and therefore the resolution of particles in the denoised images. But these effects are not critical to the performance of the FP-NLM filter. We would also like to note that Haar-like features can equally be used to classify dark particles of interest against a bright background (Yu and Bajaj, 2004; Zhu et al., 2003) by simply changing the sign in Eq. (1), which may have further applications in biology and other fields.

Robust detection of features of interest is a critical step in the automation of particle tracking which in turn is critical for the analysis of complex biological data. We find that by using our FP-NLM filter we are able to reduce the illumination dosage level by a factor of almost five times and still achieve reliable feature detection. This should enable increased, more biologically relevant imaging rates and/or durations. We believe that FP-NLM can have significant benefits in many situations where quantitative analysis of dynamic components is required to fully investigate the molecular processes involved. Quantitative analysis of the denoised images of EB1 tracks will allow us to map both the distribution and location of MT to aid our understanding of how transport processes are organized and regulated within cells and tissues.

## Figures and Tables

**Table 1:** Parameters of FP-NLM filter [used in Eqs. (1)–(6)].

Parameter		Values
*W*_***i***_	Searching window centered at position ***i***, used to calculate the average in Eq. (5)	21 × 21 pixels used in FP-NLM and NLM. Variable window size in PBF
*N*_***i***_	Neighborhood centered at position ***i***, used to calculate the Euclidean distance	7 × 7 pixels used in FP-NLM and NLM; 9 × 9 pixels used in PBF
*h*	First filtering parameter	*h* = *α*_1_ · *σ*,*α*_1_ ∈ [0.75,1] chosen in NLM (Buades et al., 2008), *h* = 0.9*σ* used in FP-NLM; N/A for PBF
*g*	Second filtering parameter	*g* = *σ*/*α*_2_, *α*_2_ ∈ [50,100] is chosen empirically in FP-NLM; N/A for NLM and PBF. Reduce to improve detection of weak features
*s*	The scale of Haar window used to calculate Haar-like features $H_{k}^{s}$ (*k* = 1, 2, 3)	Sufficiently large to cover the size range of particle; *s* = 1–5 is used in the paper, from 7 × 7 to 37 × 37 pixels
*λ*	Weak threshold to classify each pixel into two classes (particle/background)	Typically 20% of the difference of the averaged pixel gray values between a typical particle region and a typical background region in the image
*A*_***i***_	An area centered at position ***i*** to calculate particle probability	Typically the size of the smallest particles to be detected in the image; 5 × 5 used in the paper

**Table 2:** PSNR performance comparison of denoising algorithms.^a^

RAW *σ*	NAD	TV	WF	BF	NLM	PBF	FP-NLM
20	34.26	34.81	34.05	32.01	33.44	36.04	36.59
30	31.87	32.45	32.10	28.36	31.30	33.84	34.25

a PSNR was determined from the denoised synthetic image data for the 7 different algorithms compared. Two levels of noise were tested: added Gaussian noise levels of *σ* = 20 (as shown in Fig. 2A) and 30. PSNR values of the unprocessed images were 21.88 and 18.65, respectively.
